# Intracellular annexin A2 regulates NF-*κ*B signaling by binding to the p50 subunit: implications for gemcitabine resistance in pancreatic cancer

**DOI:** 10.1038/cddis.2014.558

**Published:** 2015-01-22

**Authors:** H Jung, J S Kim, W K Kim, K-J Oh, J-M Kim, H J Lee, B S Han, D S Kim, Y S Seo, S C Lee, S G Park, K-H Bae

**Affiliations:** 1 Department of Biological Sciences, Korea Advanced Institute of Science and Technology (KAIST), Daejeon, 305-701, Republic of Korea; 2 Functional Genomics Research Center, Korea Research Institute of Bioscience and Biotechnology (KRIBB), Daejeon, 305-806, Republic of Korea; 3 Department of Pathology, College of Medicine, Chungnam National University, Daejeon, 301-721, Republic of Korea; 4 Department of Internal Medicine, Chungnam National University, Daejeon, 301-721, Republic of Korea; 5 Department of Pathology, Samsung Medical Center, Sungkyunkwan University School of Medicine, Seoul 135-710, Republic of Korea

## Abstract

Annexin A2 (ANXA2) expression is highly upregulated in many types of cancer. Although cell surface localization of ANXA2 has been reported to have a critical role in the progression and metastasis of a variety of tumors, including pancreatic cancer, the biological role of intracellular ANXA2 is not fully understood. Herein the role of intracellular ANXA2 was investigated in a pancreatic cancer cell line. We first determined whether ANXA2 is involved in NF-*κ*B signaling pathways. ANXA2 bound to the p50 subunit of NF-*κ*B in a calcium-independent manner, and the ANXA2–p50 complex translocated into the nucleus. Furthermore, ANXA2 increased the transcriptional activity of NF-*κ*B in both the resting and activated states and upregulated the transcription of several target genes downstream of NF-*κ*B, including that encoding interleukin (IL)-6, which contributes to anti-apoptotic signaling. In Mia-Paca2 cells, we determined the effects of wild-type ANXA2 and an ANXA2 mutant, Y23A, which suppresses the cell surface localization, on upregulation of NF-*κ*B transcriptional activity and secretion of IL-6. Both wild-type and Y23A ANXA2 induced anti-apoptotic effects in response to treatment with tumor necrosis factor-*α* or gemcitabine. Based on these results, we suggest that ANXA2 mediates resistance to gemcitabine by directly increasing the activity of NF-*κ*B. Collectively, these data may provide additional information about the biological role of ANXA2 in pancreatic cancer and suggest that ANXA2 is a potential biomarker for the drug resistance phenotype and a candidate therapeutic target for the treatment of pancreatic cancer.

Annexin A2 (ANXA2) is a negatively charged phospholipid-binding protein that has a functional N-terminal domain with binding sites for p11 protein (S100A10) and tissue-type plasminogen activator (tPA) and a C-terminal domain with binding sites for calcium, phospholipids, and the actin cytoskeleton. *ANXA2* is a pleiotropic gene that functions in multiple pathways, including those involved in signal transduction, membrane fusion, cell adhesion, DNA synthesis, cell proliferation, and fibrinolysis.^1, 2, 3^ In cancer, ANXA2 is overexpressed in many types of tumors, including those arising in the breast, liver, prostate, and pancreas.^4, 5^ ANXA2 has important roles in cancer cell migration, adhesion, invasion, and metastasis. Moreover, ANXA2 appears to be involved in the drug resistance phenotype of cancer cells based on the finding that ANXA2 is upregulated during acquisition of the multi-drug resistance phenotype in breast cancer cells. ANXA2 exists as a 36-kDa monomeric protein in the cytoplasm and can also form a heterotetramer containing two ANXA2 monomers and two p11 subunits. ANXA2 heterotetramers are localized on the cell surface of endothelial cells and in several types of cancers. ANXA2 heterotetramers bind to tPA to mediate the activation of plasminogen to plasmin, which facilitates degradation of the extracellular matrix and proteolytic activation of inactive proteases, such as matrix metalloproteases, leading to increased angiogenesis, migration, invasion, and metastasis of tumor cells. However, the pathophysiological role of the intracellular ANXA2 monomer is poorly understood.

Expression of ANXA2 is upregulated in pancreatic cancer cell lines and primary pancreatic tumors.^6, 7, 8^ Furthermore, Akt/mTOR signaling is responsible, at least in part, for the upregulation of ANXA2 observed in recurrent pancreatic cancer following adjuvant therapy with gemcitabine. It was thus suggested that ANXA2 may be a predictive marker for recurrence in this setting.^7, 9^ We have recently demonstrated that ANXA4 regulates the transcriptional activity of NF-*κ*B by binding to the p50 subunit of NF-*κ*B in a calcium-dependent manner.^10^ NF-*κ*B is a transcription factor that acts as an essential regulator of innate and adaptive immune responses, inflammation, apoptosis, cell survival, proliferation, and angiogenesis.^11, 12^ The five NF-*κ*B family members, such as NF-*κ*B1 (p50/p105), NF-*κ*B2 (p52/p100), RelA (p65), RelB, and c-Rel, have been identified. In resting states, NF-*κ*B is sequestered in the cytosol in an inactive form. After activation by diverse stimuli, NF-*κ*B translocates into the nucleus, where it induces transcription of a number of downstream target genes. NF-*κ*B is constitutively activated in many types of tumors, and increased NF-*κ*B activity is a hallmark of cancer.^13, 14^ Moreover, NF-*κ*B activation may be involved in the acquisition of drug resistance in pancreatic cancer, because the activity of NF-*κ*B is significantly higher in gemcitabine-resistant pancreatic cancer cells than in those that are sensitive to gemcitabine.^15^ Recent reports have shown that ANXA2 accumulates in the nuclei of prostate cancer cells and in metastatic lymph nodes in colon cancer.^16, 17^ Unlike the ANXA2 heterotetramers present on the cell surface, the biological role of nuclear ANXA2 is not fully understood.

Among the 12 ANX family members, several ANX members have been reported to interact with NF-*κ*B. However, the biological role of intracellular ANXA2 monomers in association with NF-*κ*B signaling pathways remains to be elucidated. In the present study, we demonstrate for the first time that ANXA2 upregulates the transcriptional activity of NF-*κ*B by binding to and facilitating nuclear translocation of the p50 subunit of NF-*κ*B. In addition, overexpression of ANXA2 is linked to gemcitabine resistance in pancreatic carcinoma through NF-*κ*B-dependent expression of several anti-apoptotic genes.

## Results

### ANXA2 interacts with the p50 subunit of NF-*κ*B in a calcium-independent manner

**Figure 1:**
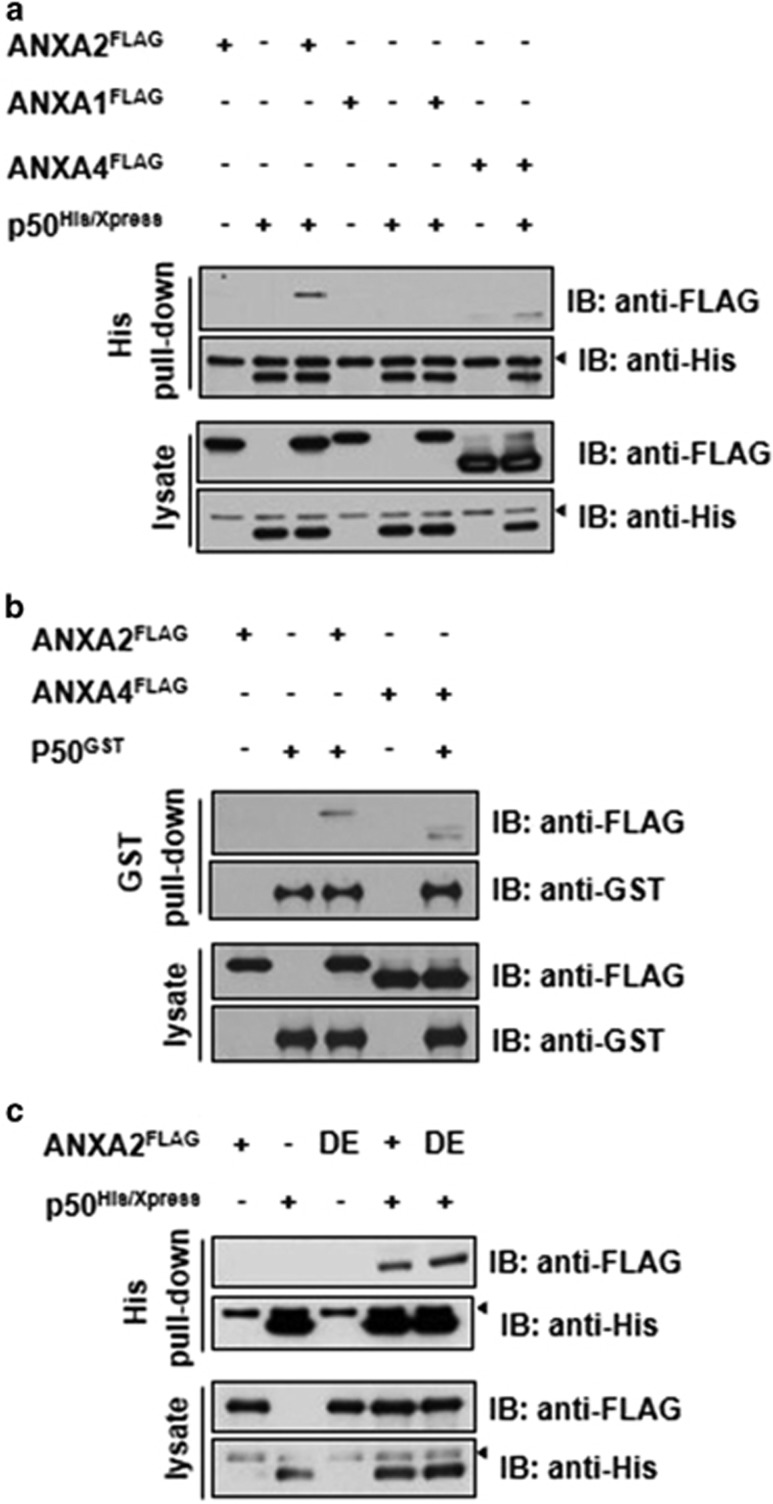
ANXA2 interacts with the p50 subunit of NF-*κ*B in a Ca^2+^-independent manner. (**a**) Interaction between ectopic ANXA2 and p50 in HEK-293 cells. His/Xpress-tagged p50 was pulled down using Ni-NTA affinity agarose beads, and pellets were analyzed by western blotting with the indicated antibody. ‘◂' indicates a non-specific band. ANXA1 and ANXA4 were used as negative and positive controls, respectively. (**b**) Interaction between ectopic ANXA2 and p50 in HEK-293 cells. GST-tagged p50 was pulled down using glutathione affinity beads and analyzed by western blotting with the indicated antibody. FLAG-tagged ANXA4 was used as the positive control. (**c**) To determine whether Ca^2+^ was essential for the interaction between ANXA2 and p50, an ANXA2 D/E mutant was constructed in which the Ca^2+^-binding sites were inactivated by replacing Asp or Glu residues with Ala. The interaction between ANXA2 D/E and p50 was measured by pull-down and western blotting analysis. ‘◂' indicates a non-specific band. IB, immunoblot

To determine possible interactions between ANXA2 and the p50 subunit of NF-*κ*B, Flag-tagged ANXA2 and His-tagged p50 proteins were overexpressed in HEK-293 cells. His pull-down and immunoblot analyses were then performed on cell extracts. Binding of ANXA2 to the p50 subunit of NF-*κ*B *in vitro* was much stronger than binding between ANXA4 and p50 (Figure 1a). Among the ANX family members, ANXA1 shows the highest amino-acid sequence homology to ANXA2, although it did not interact with p50. Binding of ANXA2 to p50 was also observed using GST pull-down and immunoblot analyses with GST-tagged p50 proteins (Figure 1b). It was next determined whether the interaction between ANXA2 and p50 is dependent on the Ca^2+^-binding ability of ANXA2. For this purpose, a D/E mutant of ANXA2 was constructed in which the Ca^2+^-binding sites were inactivated by replacing Asp or Glu residues in calcium-binding pocket with Ala. ANXA2 binding to the p50 subunit of NF-*κ*B appeared to be independent of the Ca^2+^-binding ability of ANXA2, because the wild-type and D/E mutant of ANXA2 showed a similar ability to bind p50 (Figure 1c).

### The transcriptional activity of NF-*κ*B is increased in response to ANXA2 expression

**Figure 2:**
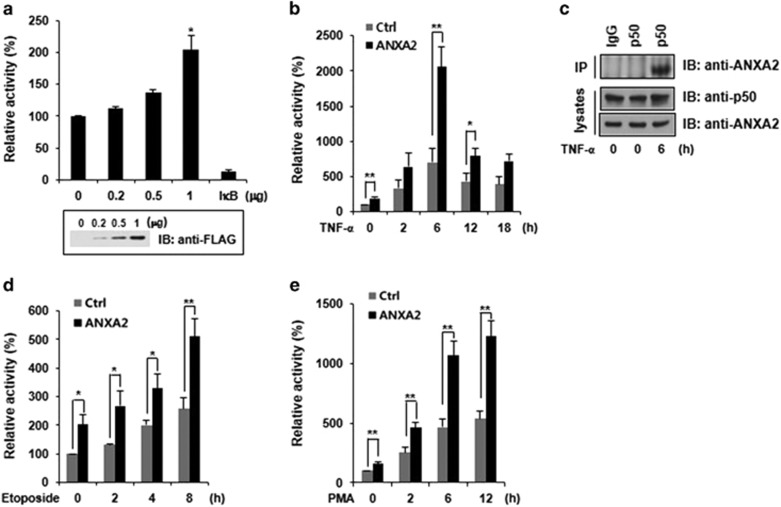
The transcriptional activity of NF-*κ*B is upregulated by ectopically expressed ANXA2. All data are representative of three independent experiments. (**a**) The indicated amounts of C-terminal FLAG-tagged ANXA2 were transfected into HeLa cells, and luciferase assays were performed. I*κ*B was used as a negative control. Expression of ectopically expressed ANXA2 was determined by western blotting analysis with an anti-FLAG antibody. (**b**) Transcriptional activity of NF-*κ*B after TNF-*α* stimulation in HeLa cells transfected with C-terminal FLAG-tagged ANXA2. (**c**) Interaction between endogenous ANXA2 and p50 in HeLa cells with or without TNF-*α* treatment. HeLa cells were treated with 10 ng/ml TNF-*α* for the indicated amount of time. Cell extracts were immunoprecipitated with anti-p50 rabbit polyclonal IgG or anti-rabbit preimmune IgG, separated by sodium dodecyl sulfate-polyacrylamide gel electrophoresis, and analyzed by western blotting with the indicated antibodies. (**d**) NF-*κ*B transcriptional activity after etoposide treatment. (**e**) NF-*κ*B transcriptional activity after PMA treatment. Error bar, S.D.; *P<0.05; **P<0.01. IB, immunoblot; IP, immunoprecipitation

To explore the effects of ANXA2 on the transcriptional activation of NF-*κ*B, a luciferase reporter assay was performed after expressing ANXA2 in HeLa cells. The N-terminal domains of ANX proteins may confer unique characteristics to each ANX family member and appear to be structurally unstable, because they are short and flexible.^18^ Therefore, we used C-terminal-tagged ANXA2 in all the experiments. Expression of ANXA2 significantly enhanced the transcriptional activity of NF-*κ*B in a dose-dependent manner (Figure 2a). Next, we determined whether ANXA2 can affect the transcriptional activity of NF-*κ*B in response to various stimuli that activate NF-*κ*B signaling pathways. First, we triggered the NF-*κ*B signaling pathway via tumor necrosis factor-*α* (TNF-*α*) stimulation and then assayed luciferase activity with or without ANXA2 expression over time (Figure 2b). The transcriptional activity of NF-*κ*B was significantly increased by ectopic expression of ANXA2 regardless of stimulation with TNF-*α*, although a stronger interaction was detected between ANXA2 and p50 in the presence of TNF-*α* than in the absence of TNF-*α* (Figure 2c). Ectopic expression of ANXA2 was also increased in the presence of etoposide and phorbol 12-myristate 13-acetate (PMA) (Figures 2d and e). These results clearly suggest that ANXA2 upregulates the transcriptional activity of NF-*κ*B via interaction with the p50 subunit of NF-*κ*B.

### The N-terminal domain of ANXA2 is essential for the interaction with p50

**Figure 3:**
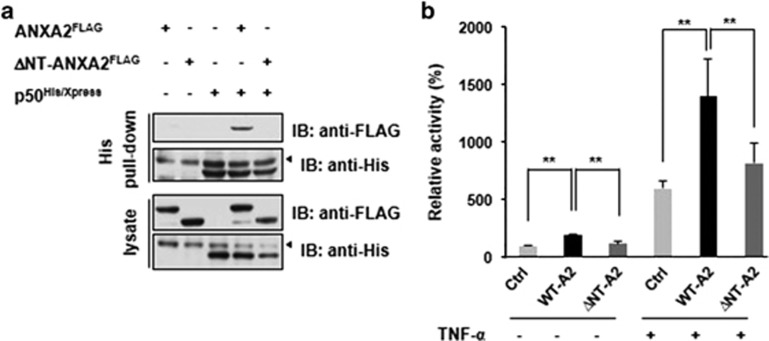
The N-terminal domain of ANXA2 is essential for the interaction with p50. (**a**) To determine whether the N-terminal domain of ANXA2 is involved in the interaction with p50, an N-terminal domain deletion mutant of ANXA2 (ΔNT-ANXA2) was constructed, and the interaction between p50 and ΔNT-ANXA2 was examined. ‘◂' indicates a non-specific band. (**b**) C-terminal FLAG-tagged ANXA2 (WT-A2) or the C-terminal tagged N-terminal deletion mutant of ANXA2 (ΔNT-A2) was transfected into HeLa cells, and the transcriptional activity of NF-*κ*B was assayed after stimulation with TNF-*α*. Data are representative of three independent experiments. Error bar, S.D. **P<0.01. IB, immunoblot

The N-terminal domains of ANX family proteins are highly variable, while the C-terminal domains are generally well conserved. Therefore, we determined whether the N-terminal domain of ANXA2 was responsible for the interaction with the p50 subunit of NF-*κ*B. Compared with wild-type ANXA2, the N-terminal deletion mutant of ANXA2 did not bind to p50 (Figure 3a) and failed to upregulate the transcriptional activity of NF-*κ*B, as determined by a luciferase assay, in either the resting state or when activated with TNF-*α* (Figure 3b). These results indicate that the N-terminal domain of ANXA2 is important for its interaction with p50 and for upregulation of the transcriptional activity of NF-*κ*B.

### ANXA2 knockdown affects the transcriptional activity of NF-*κ*B and cell viability

**Figure 4:**
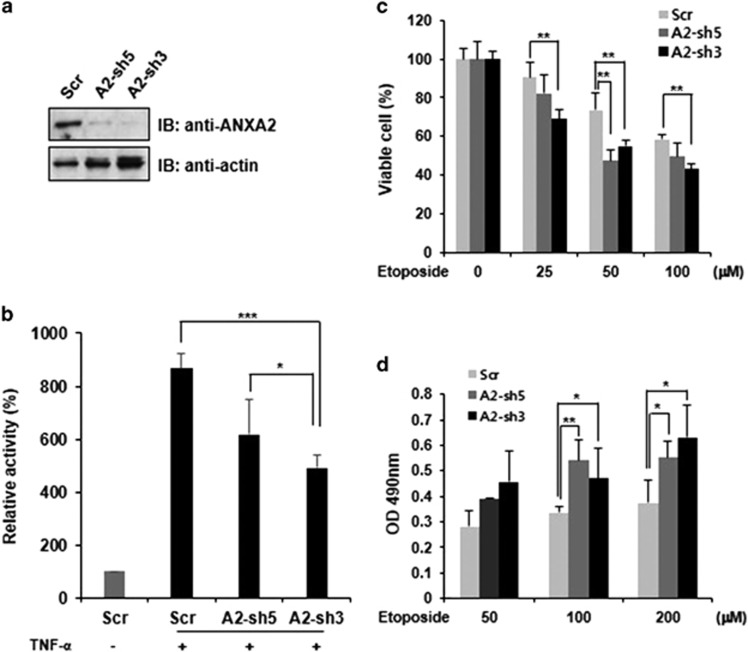
ANXA2 knockdown affects NF-*κ*B activity and decreases cell viability. (**a**) HeLa cells were infected with a retrovirus expressing scrambled control DNA (SCR), ANXA2-targeting shRNA-5 (A2-sh5), or ANXA2-targeting shRNA-3 (A2-sh3), and transduced cells were enriched by fluorescence-activated cell sorting. The knockdown of endogenous ANXA2 expression was confirmed by western blotting analysis. (**b**) NF-*κ*B transcriptional assay (luciferase reporter assay system) in ANXA2-knockdown cells after treatment with or without TNF-*α*. (**c**) Cell viability in ANXA2-knockdown cells after etoposide treatment. Data are representative of three independent experiments. (**d**) LDH cytotoxicity in ANXA2-knockdown cells after treatment with etoposide at the indicated concentrations. Data are representative of three independent experiments. Error bar, S.D. *P<0.05; **P<0.01; ***P<0.001. IB, immunoblot

To investigate the effects of decreased ANXA2 expression on the NF-*κ*B signaling pathway, a knockdown experiment using short hairpin RNA (shRNA) was performed. HeLa cells were transduced with retroviruses expressing shRNA against ANXA2 or a scrambled shRNA control, and then transduced cells were enriched by flow cytometry and cultured. Retroviral transduction of shRNA against ANXA2 substantially decreased ANXA2 expression in HeLa cells (Figure 4a). In addition, in comparison to control cells, the transcriptional activity of NF-*κ*B was significantly suppressed by nearly 50% in ANXA2-knockdown cells (Figure 4b). This decrease in transcriptional activity in response to ANXA2 knockdown was proportional to the levels of expression of ANXA2. In addition, cell viability after etoposide treatment was significantly lower in ANXA2-knockdown cells than in control cells (Figure 4c), while cellular cytotoxicity was significantly increased upon ANXA2 knockdown (Figure 4d) as determined by the lactate dehydrogenase (LDH) assay. These results suggest that ANXA2 is involved in controlling cell fate (survival and death) and resistance to cellular damage (e.g., caused by drugs and environmental factors) by altering the activity of NF-*κ*B.

### ANXA2 co-translocates into the nucleus with p50

**Figure 5:**
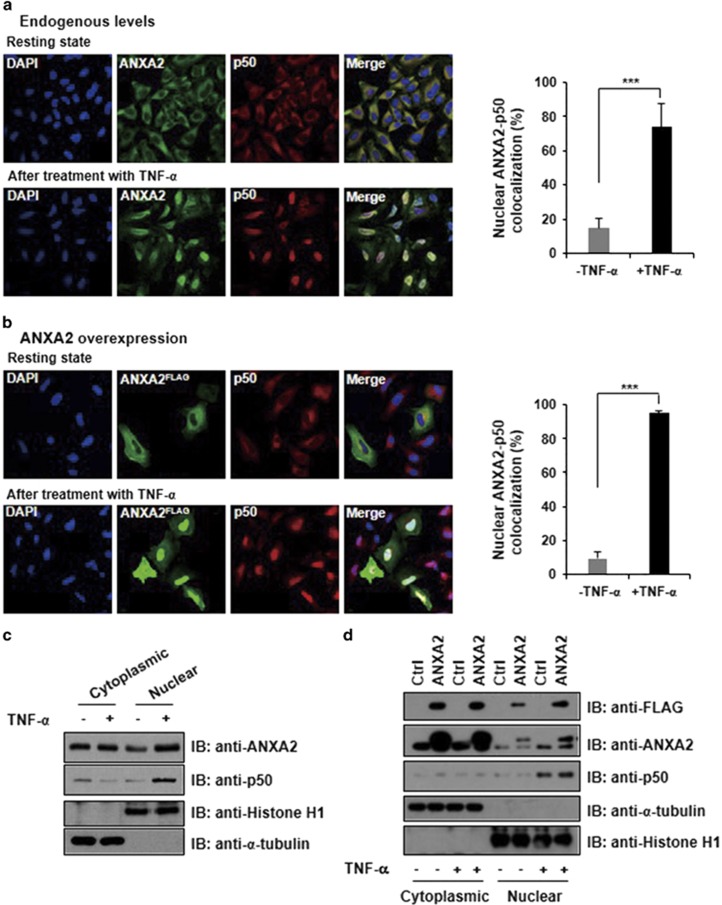
ANXA2 translocates into the nucleus together with p50 after treatment with TNF-*α*. All data are representative of three independent experiments. Error bar, S.D. ***P<0.005. (**a**) The change in the localization of endogenous p50 and endogenous ANXA2 was analyzed by immunofluorescence confocal microscopy after treatment with or without TNF-*α*. Nuclei were stained with DAPI. The nuclear colocalization of endogenous ANXA2 and p50 was quantitated as a graph. (**b**) The change in the localization of ectopically expressed ANXA2 and endogenous p50 was analyzed by immunofluorescence confocal microscopy after treatment with or without TNF-*α* (30 ng/ml) for 1 h. The nuclear colocalization of ectopically expressed ANXA2 and p50 was quantitated as a graph. (**c**) Subcellular fractionation of HeLa cell treated without or with TNF-*α*. Histone H1 and *α*-tubulin serve as nuclear and cytosol-specific marker, respectively. (**d**) HeLa cells containing vector control or vector expressing ANXA2 were treated without or with TNF-*α* (30 ng/ml), and then the cytosol and nucleus fractions were prepared by differential centrifugation. Histone H1 and *α*-tubulin serve as nuclear and cytosol-specific marker, respectively

The subcellular localizations of ANXA2 and p50 were monitored in the resting and stimulated states using confocal microscopy and subcellular fractionation. Endogenous ANXA2 and p50 co-translocated into the nucleus upon treatment with TNF-*α*, while a small amount of endogenous ANXA2 was observed in the nucleus in the resting state (Figure 5a). Similarly, a small proportion of ectopically expressed ANXA2 and endogenous p50 localized to the nucleus in the resting state, whereas most ectopically expressed ANXA2 and endogenous p50 translocated into the nucleus in response to TNF-*α* (Figure 5b). This indicates that both endogenous and ectopically expressed ANXA2 translocate into the nucleus together with the NF-*κ*B p50 subunit, where it may activate the expression of target genes downstream of NF-*κ*B. Subcellular fractionation data also demonstrated that ANXA2 and p50 translocated from the cytoplasm to the nucleus after treatment with TNF-*α* (Figures 5c and d).

### ANXA2 induces the expression of several genes related to anti-apoptotic signaling by interacting with the NF-*κ*B p50 subunit

**Figure 6:**
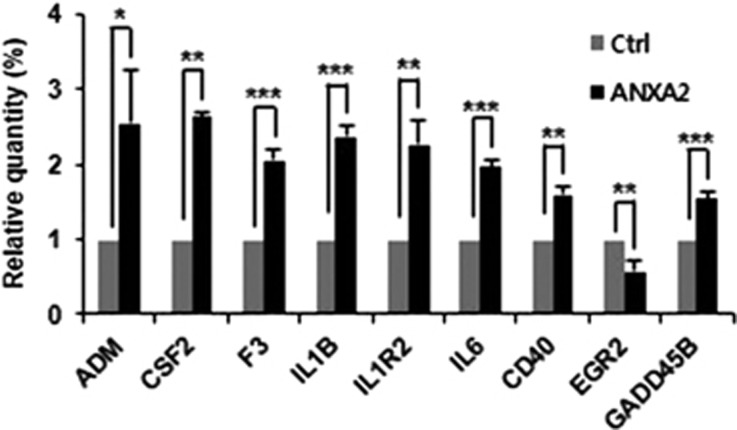
Changes in the expression of NF-*κ*B downstream target genes in response to ectopic ANXA2. To ascertain the changes in NF-*κ*B downstream target gene expression in response to ectopic ANXA2, HeLa cells transfected with C-terminal FLAG-tagged ANXA2 were analyzed using a quantitative PCR array of NF-*κ*B downstream target genes. The expression of several NF-*κ*B target genes was significantly altered. Error bar, S.D. All data are representative of three independent experiments. *P<0.05; **P<0.01; ***P<0.005

To identify genes induced by ANXA2-mediated transcriptional upregulation of NF-*κ*B, a qPCR array experiment was performed in HeLa cells expressing ANXA2 and treated with TNF-*α*. In cells overexpressing ANXA2, among the 84 NF-*κ*B target genes, expression of adrenomedullin, CSF2 (GM-CSF), F3, IL-1B, IL1R2, IL-6, CD40, and GADD45B was significantly increased, whereas that of EGR2 was significantly decreased (Figure 6). Many of these genes are related to cell survival, anti-apoptotic signaling, and the drug resistance phenotype in cancer, although the expression of several positive regulators of apoptosis did not change as determined by qPCR array data (Supplementary Figure S1). Thus, based on these results, we suggest that overexpression of ANXA2 increases the expression of anti-apoptotic genes, leading to an anti-apoptotic or drug-resistant phenotype.

### Overexpression of ANXA2 may be involved in gemcitabine resistance in pancreatic cancer cells

**Figure 7:**
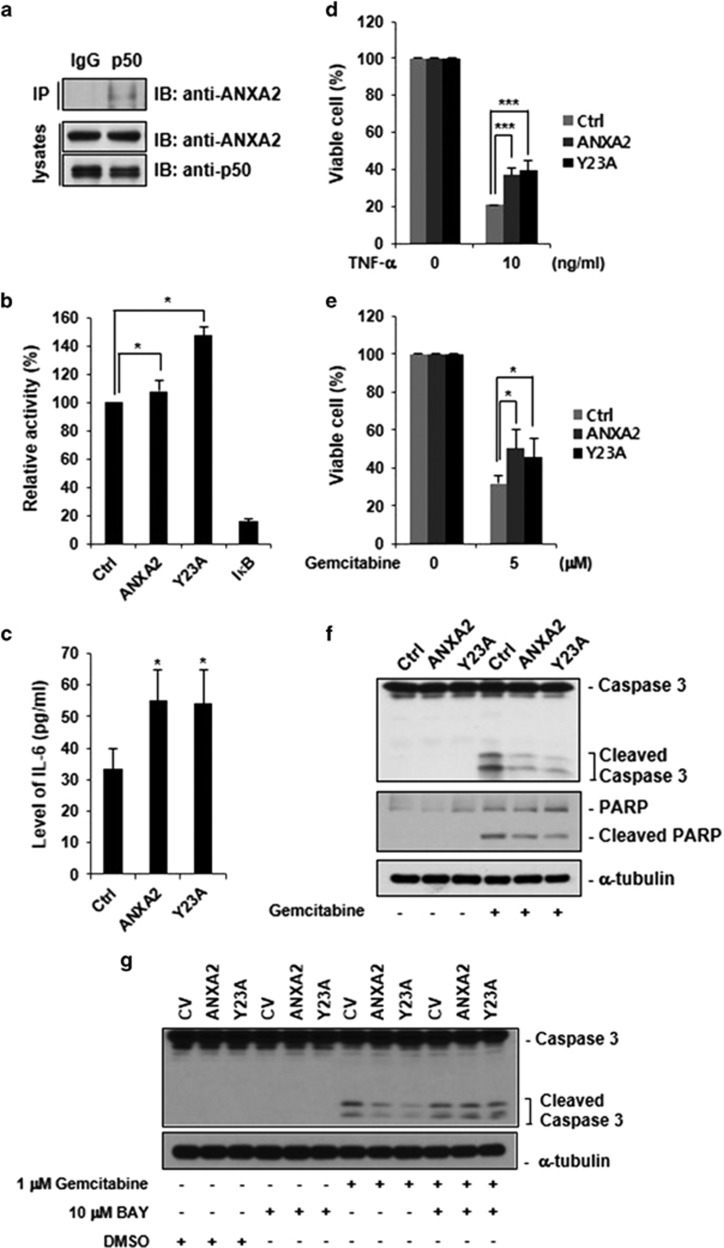
Functional analysis of the interaction between ANXA2 and p50 in Mia-Paca2 cells. All data are representative of three independent experiments. (**a**) Interaction between endogenous ANXA2 and p50 in Mia-Paca2 cells. Cell extracts were immunoprecipitated with anti-p50 rabbit polyclonal IgG or anti-rabbit preimmune IgG, separated by sodium dodecyl sulfate-polyacrylamide gel electrophoresis, and analyzed by western blotting with the indicated antibodies. (**b**) Mammalian expression vectors containing the indicated DNA sequences were introduced into Mia-Paca2 cells by electroporation, and a NF-*κ*B transcriptional activity assay was performed. (**c**) IL-6 secretion into the culture medium by cells expressing wild-type or Y23A ANXA2 assessed by an enzyme-linked immunosorbent assay. (**d**) Viability (WST reagent) of Mia-Paca2 cells expressing wild-type or Y23A ANXA2 after treatment with TNF-*α* for 48 h. (**e**) Viability (WST reagent) of Mia-Paca2 cells expressing wild-type or Y23A ANXA2 after treatment with gemcitabine treatment for 48 h. (**f**) Caspase 3 levels and poly ADP-ribose polymerase (PARP) cleavage were confirmed by western blotting analysis after gemcitabine treatment (5 *μ*M) for 48 h. (**g**) One day before gemcitabine treatment, Mia-Paca2 cells expressing wild-type or Y23A ANXA2 (7 × 10^5^ cells) were plated into 100 mm cell culture plate. 1 *μ*M gemcitabine and 10 *μ*M Bay 11-7082 were treated for 36 h, and cleaved caspase 3 was confirmed by western blotting analysis. *P<0.05; ***P<0.005. IB, immunoblot; IP, immunoprecipitation

We hypothesized that the interaction between ANXA2 and p50 might be related to the accumulation of ANXA2 in the nuclei of pancreatic cancer cells. We examined whether the biological role of intracellular ANXA2 in the regulation of anti-apoptotic gene expression, via its interaction with p50, co-translocation into the nucleus, and upregulation of the transcriptional activity of the p50 subunit, is related to the drug resistance phenotype in pancreatic cancer cells. To this end, we constructed an Y23A ANXA2 mutant, which cannot translocate to the cell surface,^19^ to exclude the effects of cell surface-localized ANXA2. Both wild-type and Y23A ANXA2 bound to p50 and upregulated the transcriptional activity of NF-*κ*B (Supplementary Figure S2). Mia-Paca2 cells exhibited the lowest levels of ANXA2 expression among several pancreatic cancer cell lines tested (Supplementary Figure S3); therefore, we constructed stable Mia-Paca2 human pancreatic carcinoma cell lines expressing wild-type or Y23A ANXA2 using a retroviral gene expression system (Supplementary Figure S4). We first determined that there was an interaction between endogenous ANXA2 and p50 in Mia-Paca2 cells (Figure 7a). The transcriptional activity of NF-*κ*B was also increased following the expression of wild-type or Y23A ANXA2; notably, Y23A ANXA2 induced NF-*κ*B activity more robustly than wild-type ANXA2 (Figure 7b). Furthermore, IL-6 secretion was increased upon ectopic expression of wild-type or Y23A ANXA2 (Figure 7c). The effects of wild-type and Y23A ANXA2 on TNF-*α*-induced apoptosis of Mia-Paca2 cells were then determined using a cell viability assay. The viability of cells expressing wild-type or Y23A ANXA2 was two-fold higher than that of control cells (vector alone) (Figure 7d). To examine the role of ANXA2 in the drug resistance phenotype of pancreatic cancer cells, the viability of Mia-Paca2 cells was assessed after treatment with gemcitabine at the indicated concentrations for 48 h. After treatment with gemcitabine, the numbers of viable Mia-Paca2 cells were significantly higher among those ectopically expressing wild-type or Y23A ANXA2 than among control cells (Figure 7e). We also analyzed cleavage of caspase 3 and PARP to validate the anti-apoptotic effects of ANXA2. Levels of cleaved caspase 3 (activated caspase 3) and cleaved PARP were lower in cells expressing wild-type or Y23A ANXA2 than in control cells (Figure 7f). On the other hand, the treatment with BAY 11-7082 (NF-*κ*B-specific inhibitor) recovered the effects of wild-type or Y23A ANXA2 on cleavage of caspase 3 (Figure 7g), indicating that ANXA2 induces drug resistance against gemcitabine through the activation of NF-*κ*B signaling pathway. To determine the possible molecular mechanism underlying ANXA2-induced gemcitabine resistance, we examined the expression of major drug resistance-related genes, such as MDR1 (multi-drug resistance protein 1), MRP (multi-drug resistance-associated protein), GST4A (glutathione *S*-transferase induced by drugs and toxins), and LRP (lung-resistance protein) in the control, ANXA2, and Y23A ANXA2 expressing Mia-Paca2 cells after the treatment of gemcitabine. However, there was no significant changes in the expression of those genes induced by ANXA2 or Y23A ANXA2 expression (data not shown). Collectively, these results clearly suggest that gemcitabine resistance was induced by overexpression of ANXA2 and was linked to the interaction between ANXA2 and p50, which subsequently upregulated anti-apoptotic NF-*κ*B target genes in pancreatic cancer cells.

## Discussion

Tumor cells are highly heterogeneous and genetically unstable; therefore, anti-cancer therapies are often of limited benefit due to the inevitable emergence of acquired drug resistance. Indeed, resistance to chemotherapy is believed to be responsible for treatment failure in >90% of patients with metastatic cancers.^20^ New treatments to overcome drug resistance would have undoubted impact on patient survival. However, at present, understanding of the mechanisms involved in the development of drug resistance is incomplete. In this regard, more in-depth understanding of the molecular and biochemical mechanisms underlying drug resistance is needed to develop novel and effective therapies for patients with cancer.

Among the known mechanisms of drug resistance, evasion of apoptosis is a key feature of acquired drug resistance in tumor cells.^20^ In this study, intracellular ANXA2 monomers induced the expression of several anti-apoptotic gene products, such as IL-6, leading to the resistance of human pancreatic cancer cells to gemcitabine through the interaction with the p50 subunit of NF-*κ*B and subsequent increases in the transcriptional activity of NF-*κ*B. IL-6 is responsible for drug resistance and anti-apoptotic signaling in prostate cancer cells by its induction of STAT3 and bcl-xL.^21^ In addition, numerous reports have shown that IL-6 has an anti-apoptotic role in gastric and cervical cancer by upregulating Mcl-1.^22, 23^ Recently, Inokuchi *et al.*^16^ reported that ANXA2 contributes to cell proliferation and IL-6 secretion in prostate cancer. Secretion of IL-6 is dramatically decreased in prostate cancer cells upon ANXA2 knockdown, while overexpression of naive ANXA2 increases IL-6 secretion. However, further experiments to investigate the molecular mechanism underlying ANXA2-dependent IL-6 secretion were not performed in this previous study.

Herein we demonstrated that ANXA2-mediated transcription of IL-6 mRNA and IL-6 protein secretion were significantly increased through the direct interaction of ANXA2 with p50 and their co-translocation into the nucleus. Preliminary immunohistochemical staining showed increased expression of ANXA2 and p50 (although their distributions were sparse) and their nuclear co-localization in primary pancreatic cancers (data not shown). ANXA2-dependent IL-6 expression was also demonstrated in plasmin-treated human macrophages.^24^ However, our data appear to reflect the effects of intracellular ANXA2, because cells expressing wild-type ANXA2 or the Y23A ANXA2 mutant, which cannot translocate to the cytoplasmic membrane, showed similar levels of IL-6 secretion. This suggests that intracellular ANXA2 monomers regulate the activity of NF-*κ*B and enhance IL-6 secretion via a direct interaction with the p50 subunit of NF-*κ*B.

NF-*κ*B is constitutively activated in many types of cancer and activated NF-*κ*B can have critical roles in the progression of cancer through regulation of cell survival, apoptosis, and drug resistance.^25, 26^ It was recently reported that the interaction between alternatively spliced segments of tenascin-C and ANXA2 on the cell surface of pancreatic cancer cells induces gemcitabine resistance through canonical PI3K/Akt/NF-*κ*B signaling pathways,^27^ although the precise mechanism by which ANXA2 and tenascin-C interact to affect gemcitabine resistance is unknown. In addition, several reports have indicated that cell surface-localized ANXA2 can bind to progastrin and gastrin-like peptides and that these proteins then activate NF-*κ*B signaling and mediate anti-apoptotic effects in colon cancer cells, pancreatic cancer cells, and intestinal epithelial cells.^2, 28, 29, 30^ However, our data are the first to report that the intracellular ANXA2 monomer can directly regulate NF-*κ*B signaling pathways.

Although other ANX family members also mediate the regulation of NF-*κ*B signaling and biochemical interactions and signaling networks involved in the regulation of NF-*κ*B, these pathways appear to be complex and specific for each ANX family member. ANXA4 and ANXA2 interact with the p50 subunit of NF-*κ*B, while ANXA1 and ANXA6 bind to the p65 subunit of NF-*κ*B.^10, 31, 32^ We previously reported that binding of ANXA4 to the p50 subunit of NF-*κ*B is dependent upon the levels of intracellular Ca^2+^. However, in the current study, ANXA2 bound to the p50 subunit of NF-*κ*B in a Ca^2+^-independent manner, despite ANXA2 having the highest affinity for Ca^2+^ among the ANX family members.^33^ Therefore, more information is required to understand these observations in the context of the entire signaling network involving each member of the ANX family and NF-*κ*B proteins.

ANXA2 has a critical role in the progression and metastasis of a variety of tumors, and it is therefore considered to be a potential therapeutic target in cancer. In this regard, several investigations have suggested the feasibility of such an approach. Antibodies against ANXA2 and angiostatin, an anti-angiogenic protein, block the generation of plasmin mediated by ANXA2 on the surface of endothelial cells and tumor cells and show potent anti-angiogenic and anti-tumor effects.^34, 35^ Polymeric nanoparticles coupled with an ANXA2-targeting siRNA vector inhibit prostate cancer growth in mice.^36^ A polypeptide with tumor-targeting and anti-angiogenic effects, TM601, was recently shown to inhibit the activation of plasminogen to plasmin by binding to ANXA2 in endothelial cells and in glioma, lung, and pancreatic cancer cells.^37^ Most of these approaches target ANXA2 proteins localized on the cell surface. However, according to our observations, tumor cells can trigger NF-*κ*B signaling through intracellular ANXA2 and may escape apoptosis by inducing the expression of anti-apoptotic proteins. Therefore, future therapeutic approaches targeting ANXA2 should consider intracellular ANXA2, in addition to cell surface-localized ANXA2.

The finding that TNF-*α* can stimulate ANXA2-dependent secretion of IL-6 in tumor cells suggests that ANXA2 can also modulate the tumor microenvironment. For instance, Kim *et al.* demonstrated that tumor-associated macrophages promote prostate cancer bone metastasis through the production of IL-6, which in turn recruits more macrophages to the tumor site that produce further TNF-*α*, leading to increased production of IL-6 by tumor cells.^38^ The role of tumor-associated macrophages in the progression of pancreatic cancer has been well documented.^39^ Accordingly, the biological importance and clinical relevance of the present findings warrant further *in vivo* evaluation.

In summary, intracellular ANXA2 can directly regulate the transcriptional activity of NF-*κ*B by binding to the p50 subunit of NF-*κ*B, inducing gemcitabine resistance in pancreatic cancer cells through upregulation of anti-apoptotic genes, including that encoding IL-6. Collectively, our data strongly suggest that ANXA2 is a good biomarker to predict patient outcomes after therapy with gemcitabine and may be a useful therapeutic target for pancreatic cancer.

## Materials and Methods

### Cell lines, reagents, and plasmids

HeLa, HEK-293, SW480, and Mia-Paca2 cells were from the American Type Culture Collection (ATCC; Rockville, MA, USA). Media and other cell culture reagents were from Gibco BRL (Grand Island, NE, USA). The following reagents were used: TNF-*α* (Sigma, St. Louis, MO, USA), PMA (Sigma), Etoposide (Sigma), Gemcitabine hydrochloride (Sigma), and Bay 11-7082 (Santa Cruz Biotechnology, Santa Cruz, CA, USA). The following antibodies were used: mouse anti-FLAG (Sigma), rabbit anti-GST (Sigma), rabbit anti-actin (Sigma), mouse anti-His (Millipore, Temecula, CA, USA), goat anti-ANXA2 (GeneTex, Irvine, CA, USA), rabbit anti-NF-*κ*B p50 (Cell Signaling, Danvers, qMA, USA), rabbit anti-caspase-3 (Cell Signaling), and rabbit anti-PARP (Cell Signaling). Genes encoding C-terminal FLAG-tagged human ANXA2, ANXA1, and ANXA4 were constructed by PCR, followed by cloning into the pcDNA3.1/Zeo plasmid and the pRetroX-IRES-ZsGreen1 vector. The Xpress/His-tagged p50 expression plasmids were generated by PCR and subcloned into pcDNA4/HisMax (Invitrogen, Carlsbad, CA, USA). GST-tagged p50 was constructed in the pEBG plasmid for protein expression. The cFLAG-ANXA2 D/E mutant (D161A, D246A, D321A) expression plasmid was generated by PCR using wild-type cFLAG-ANXA2 as the template. The N-terminal deletion mutant of ANXA2 (lacking amino acids 1−23) was generated by PCR and cloned into pcDNA3.1/Zeo. The cFLAG-ANXA2 Y23A mutant was generated by site-directed mutagenesis of pRetroX-IRES-ZsGreen1-cFLAG-ANXA2 and pcDNA3.1/Zeo-cFLAG-ANXA2.

### His-tagged protein pull-down

His-tagged p50 and cFLAG-tagged ANXA2 were cotransfected into HEK-293 cells using Lipofectamine 2000 reagent (Invitrogen). Cells were lysed in Ni-NTA lysis buffer (20 mM NaH_2_PO_4_, 300 mM NaCl, 5 mM imidazole, and 0.05% Tween 20), and lysates were incubated at 4 °C for 30 min. After centrifugation at 13 000 r.p.m. for 20 min, cell lysates containing His-p50 and cFLAG-ANXA2 were mixed with Ni-NTA-agarose beads (Qiagen, Valencia, CA, USA) at 4 °C overnight with rotation. Non-specifically bound proteins were removed by washing with wash buffer. Bound proteins were eluted with 1 × SDS-PAGE sample buffer containing 250 mM imidazole and separated by 12% SDS-PAGE followed by western blotting analysis with the indicated antibodies.

### GST-tagged protein pull-down

GST-tagged p50 and cFLAG-tagged ANXA2 were cotransfected into HEK-293 cells using the Lipofectamine 2000 reagent. Cells were lysed in NP-40 lysis buffer (20  Tris, 137 mM NaCl, 1 mM EDTA, 10% glycerol, and 1% NP-40) containing protease inhibitors, and cell lysates containing GST-p50 and cFLAG-ANXA2 were mixed with Glutathione HiCap Matrix (Qiagen) overnight at 4 °C with rotation. Non-specifically bound proteins were removed by washing with wash buffer. Bound proteins were eluted with elution buffer (50 mM Tris, 150 mM NaCl, 10% glycerol, and 10% glutathione) for 30 min, and the eluted samples were separated by 12% SDS-PAGE followed by western blotting analysis with the indicated antibodies.

### Immunoprecipitation

Five microliters of IgG as a control or anti-p50 antibody were preincubated with protein A/G agarose beads at 4 °C for 3 h. Total protein lysates in lysis buffer (20 mM Tris, 137 mM NaCl, 1 mM EDTA, 10% glycerol, and 1% NP-40) containing protease inhibitors were incubated at 4 °C overnight on a tube rotator with antibody-conjugated protein A/G agarose beads. The protein-agarose bead complexes were washed three times with lysis buffer. Samples were then separated by SDS-PAGE and analyzed by western blotting analysis using an anti-ANXA2 antibody.

### Luciferase reporter assay

Cells were routinely cotransfected with a TK-Renilla luciferase plasmid (Promega, Madison, WI, USA) to normalize for the transfection efficacy. The Dual Luciferase Reporter Assay Kit from Promega was used following the manufacturer's protocol. Luciferase activity was measured with a Victor X3 2030 multi-label plate reader (PerkinElmer, Eden Prairie, MN, USA). The data are represented the mean values obtained from three independent experiments.

### RNA interference and transduction

The pSIREN-RetroQ-DsRed Express retroviral vector (Clontech, Palo Alto, CA, USA) was used to knockdown the ANXA2 expression. shRNA sequences were designed by selecting a specific target sequence for the human ANXA2 gene, as described by Sigma-Aldrich (St. Louis, MO, USA). The following gene-specific sequences were used to successfully inhibit ANXA2 expression: sh-3: Top 5′-GATCCGCGGGATGCTTTGAACATTGAATTCAAGAGATTCAATGTTCAAAGCATCCCGTTTTTTG-3′, Bottom 5′-AATTCAAAAAACGGGATGCTTTGAACATTGAATCTCTTGAATTCAATGTTCAAAGCATCCCGCG-3′ and sh-5: Top 5′-GATCCGCAAGTCCCTGTACTATTATTTCAAGAGAATAATAGTACAGGGACTTGTTTTTTG-3′, Bottom 5′-AATTCAAAAAACAAGTCCCTGTACTATTATTCTCTTGAAATAATAGTACAGGGACTTGCG-3′. These top and bottom oligonucleotides were annealed and subcloned according to the manufacturer's recommendations, and a non-targeting control shRNA (scrambled control) was obtained from Sigma-Aldrich.

### Transduction using retroviral overexpression or knockdown system

Retroviruses were produced by cotransfection of a retroviral vector and the VSV-G plasmid with Lipofectamine 2000 into GP2-293 retroviral packaging cells (Clontech). At 48–72 h after transfection, media containing retroviruses were collected and passed through a 0.45-*μ*m filter. Cells were infected with retroviruses in the presence of polybrene (8 *μ*g/ml) and enriched using a FACSAria cell sorter (BD Bioscience, San Jose, CA, USA) and further maintained in growth medium.^40, 41^

### Confocal microscopy

One day before TNF-*α* treatment, HeLa cells were seeded onto glass cover slips. Cells were then treated with 30 ng/ml TNF-*α* for 1 h, followed by fixation with 4% formaldehyde and 4% sucrose for 20 min at 4 °C. After washing twice with phosphate-buffered saline (PBS), the fixed cells were permeabilized with PBS containing 0.2% Triton X-100 at 4 °C for 15 min. Permeabilized cells were washed three times with PBS and then incubated with PBS-BG (PBS containing 0.1% bovine albumin serum and 3% fetal bovine serum) for 1 h at room temperature. Cells were incubated with the indicated primary antibody diluted in PBS-BG overnight at 4 °C. After washing with PBS, cells were incubated with Alexa Fluor 488 anti-mouse IgG, Alexa Fluor 546 anti-rabbit IgG, or Alexa Fluor 488 anti-goat IgG (1 : 1000 dilution; Molecular Probes, Eugene, OR, USA) diluted in PBS-BG for 2 h at room temperature. Finally, cells were mounted in a solution containing DAPI (Vectashield, Vector Laboratories, Inc., Burlingame, CA, USA) and observed with a laser confocal microscope (Carl Zeiss, Thornwood, NY, USA).

### Subcellular fractionation

Cell pellets were resuspended with buffer A (40 mM Tris-HCl, 10 mM NaCl, 1 mM EDTA, 1 mM DTT and protease inhibitors). Resuspended cells were incubated on ice for 15 min and vortex mixed every 5 min for 5 s. Thirty microliters of 10% NP-40 was added to the cell extracts and then vigorously shaken for 10 s. After centrifugation at 12 000 r.p.m. for 10 min at 4 °C, supernatants were moved to a new tube (cytosolic fraction). Pellet was resuspended with buffer B (40 mM Tris-HCl, 420 mM NaCl, 10% glycerol, 1 mM EDTA, 1 mM DTT and protease inhibitors). Resuspended extracts were incubated on ice for 20 min and vortex mixed vigorously every 5 min for 5 s. After centrifugation at 12 000 r.p.m. for 10 min at 4 °C, supernatants were moved to a new tube (nuclear fraction).

### Cell viability assay

Either 2500 or 5000 cells/well were plated into 96-well plate and then treated with TNF-*α* or gemcitabine at the indicated concentrations for 48 h. Cell viability was determined using the EZ-Cytox Cell Viability Assay Kit (Daeil Lab Service, Seoul, Korea).

### LDH cytotoxicity

About 5000 cells/well were plated into 96-well plates and incubated with etoposide at the indicated concentrations for 48 h. LDH cytotoxicity was assayed with the CytoTox 96 Non-Radioactive Cytotoxicity Assay Kit following the manufacturer's recommendations (Promega).

### Quantitative PCR (qPCR) array

Expression profiles of NF-*κ*B target genes in ANXA2-transfected HeLa cells were analyzed by AccuPower real-time PCR (Bioneer, Daejeon, Korea) according to the manufacturer's recommendations.

### Quantification of IL-6 in culture supernatants

Cells (2.5 × 10^5^) were plated into 12-well plates, and the supernatants were collected after 5 days. Secretion of IL-6 into culture supernatants was quantified by an enzyme-linked immunosorbent assay according to the manufacturer's protocol (R&D Systems, Minneapolis, MN, USA).

### Statistical analysis

Data are presented as the mean±S.D. Statistical significance was calculated using the Student's *t*-test. A value of *P*<0.05 was considered statistically significant.

## Abbreviations

ANXA2: annexin A2
IL: interleukin
TNF-*α*: tumor necrosis factor-*α*
shRNA: short hairpin RNA
PBS: phosphate-buffered saline
PMA: phorbol 12-myristate 13-acetate
