# Supplementary figures and images for: Intracellular annexin A2 regulates NF-κB signaling by binding to the p50 subunit: implications for gemcitabine resistance in pancreatic cancer
Source: Cell Death Dis. 2015 Jan 22;6(1):e1606–. doi: 10.1038/cddis.2014.558 (PMC4669756; doi:10.1038/cddis.2014.558)

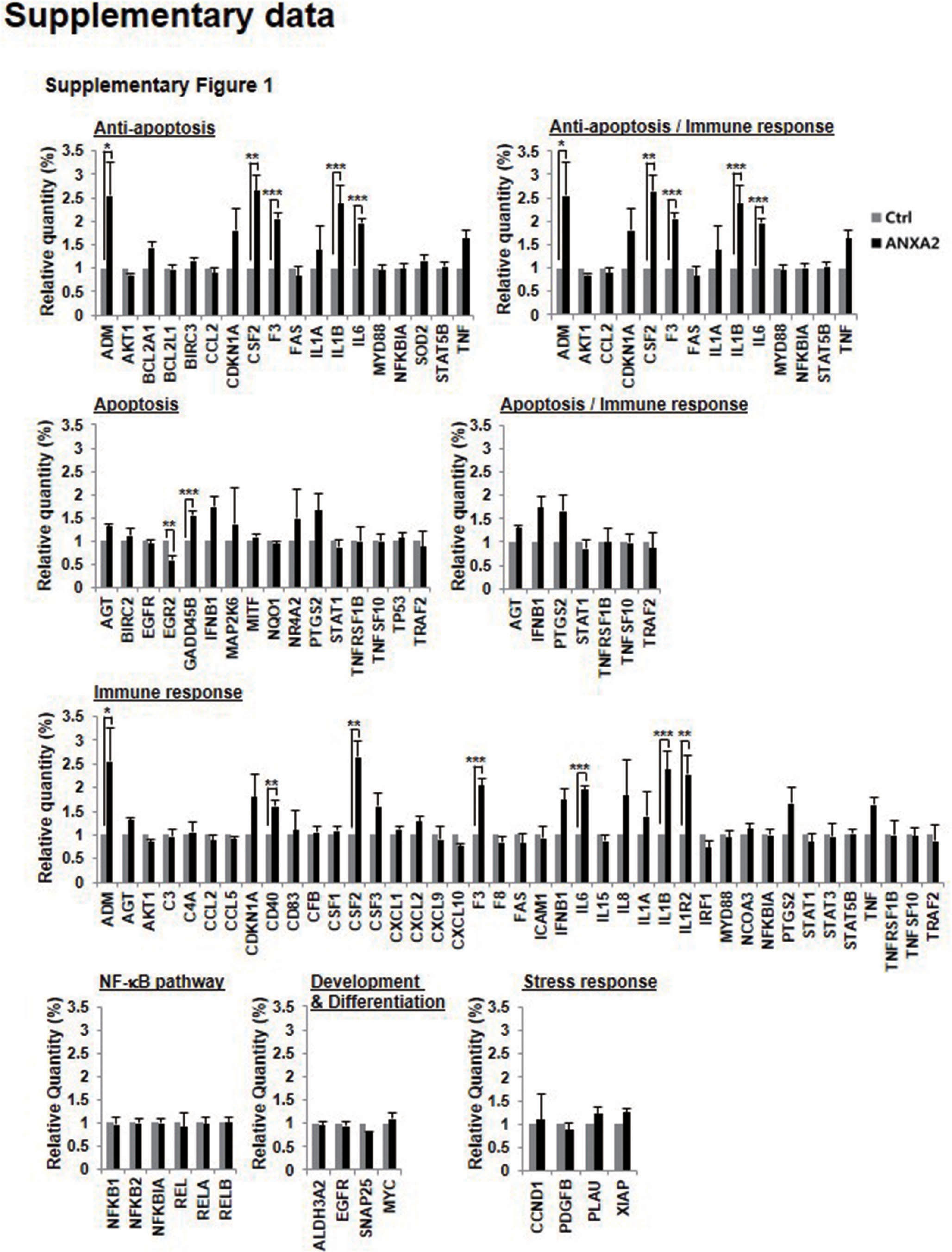

Supplement: Supplementary Figure 1 [file cddis2014558x1.tif]

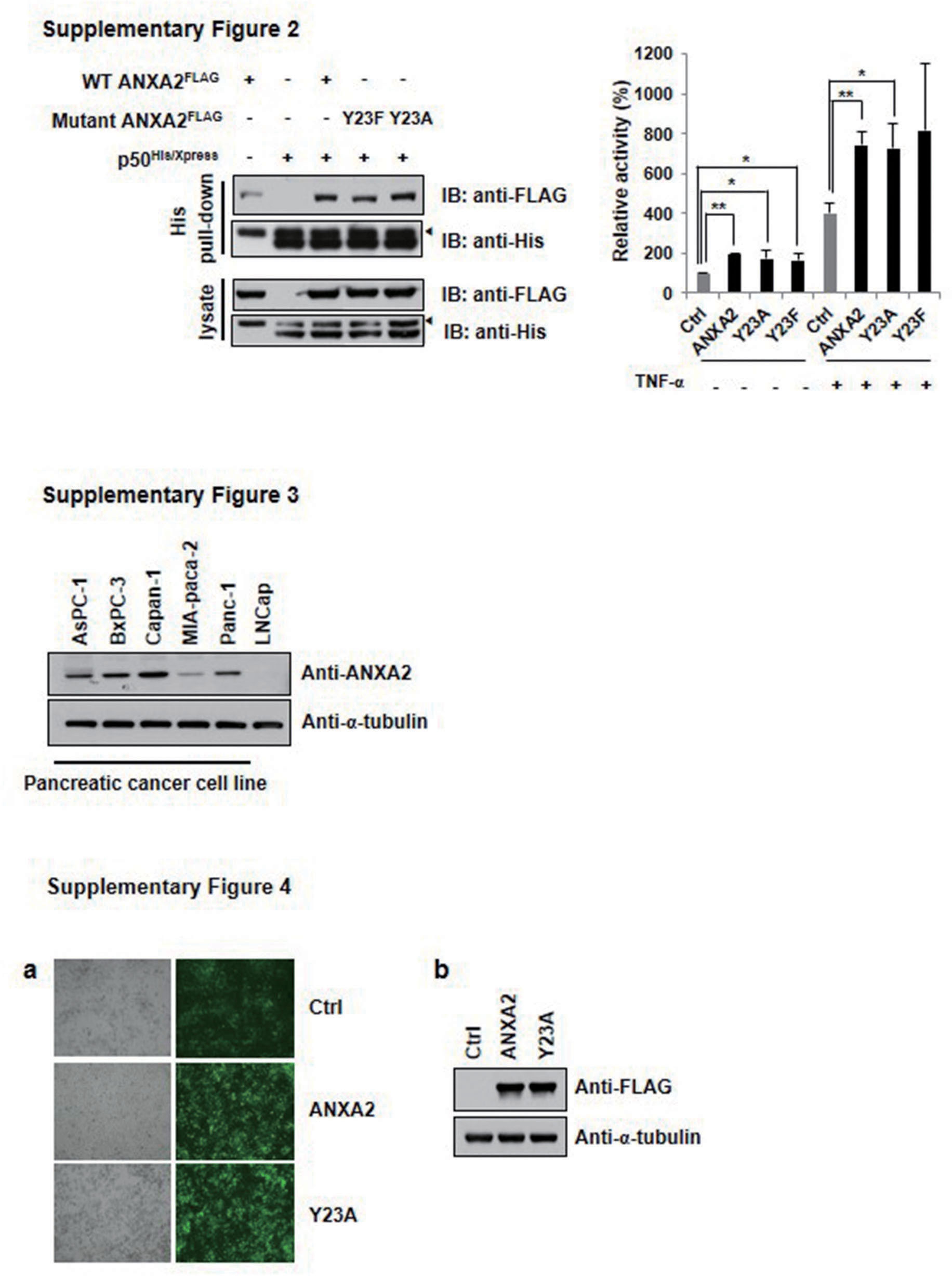

Supplement: Supplementary Figure 2, 3, 4 [file cddis2014558x2.tif]
