# Supplementary material for: Intracellular annexin A2 regulates NF-κB signaling by binding to the p50 subunit: implications for gemcitabine resistance in pancreatic cancer
Source: Cell Death Dis. 2015 Jan 22;6(1):e1606–. doi: 10.1038/cddis.2014.558 (PMC4669756; doi:10.1038/cddis.2014.558)
Supplement: Supplementary Figure Legends [file cddis2014558x3.doc]

**SUPPLEMENTARY FIGURE LEGENDS**

**Supplementary Figure 1** Classification of NF-κB downstream target genes according to their function. Functional grouping of mRNAs was performed as indicated by SABiosciences (<http://www.sabiosciences.com/rt_pcr_product/HTML/PAHS-225A.html>). Error bar, standard deviation. **P* <0.05; ***P* < 0.01; ****P* < 0.005.

**Supplementary Figure 2** Y23A ANXA2 interacts with p50 and upregulates NF-κB transcriptional activity. (**a**) Interaction between ectopic Y23A or Y23F ANXA2 (Tyr-23 phosphorylation-inactive mutants) and p50 in HEK-293 cells. His/Xpress-tagged p50 was pulled down using Ni-NTA affinity agarose beads, and the pellets were analyzed by western blotting with the indicated antibodies. “◀” indicates a non-specific band. Wild-type ANXA2 was used as a positive control. (**b**) C-terminal FLAG-tagged Y23A and Y23F ANXA2 were transfected into HeLa cells, and an NF-κB transcriptional assay was performed after stimulation with TNF-α. Error bar, standard deviation. **P* < 0.05; ***P* < 0.01. IB, immunoblot.

**Supplementary Figure 3** Mia-Paca2 as a model cell line to elucidate the functions of ANXA2 in pancreatic cancer cells. ANXA2 expression was analyzed in various pancreatic cancer cell lines. Cell lysates were separated by SDS-PAGE and western blotting was performed using an anti-ANXA2 antibody; α-tubulin was used as a loading control. The prostate cancer cell line LNCaP does not express ANXA2 and was used as a negative control.

**Supplementary Figure 4** (**a**) GFP expression in Mia-Paca2 cells assessed using fluorescence microscopy after enrichment of cells using a FACSAria cell sorter. (**b**) The ectopic expression of wild-type and Y23A ANXA2 in Mia-Paca2 cells was confirmed by western blot analysis.
